# Where There Is No Paramedic: The Sachigo Lake Wilderness Emergency Response Education Initiative

**DOI:** 10.1371/journal.pmed.1001322

**Published:** 2012-10-02

**Authors:** Aaron Orkin, David VanderBurgh, Karen Born, Mike Webster, Sarah Strickland, Jackson Beardy

**Affiliations:** 1 Division of Clinical Sciences, Northern Ontario School of Medicine, Thunder Bay, Canada; 2 Dalla Lana School of Public Health, University of Toronto, Toronto, Canada; 3 Institute of Health Policy, Management, and Evaluation, University of Toronto, Toronto, Canada; 4 Hamilton Emergency Services, Hamilton, Canada; 5 Department of Pathology and Laboratory Medicine, University of Ottawa, Ottawa, Canada; 6 Sachigo Lake First Nation, Canada

## Abstract

Aaron Orkin and colleague describe their collaboration that developed, delivered, and studied a community-based first response training program in a remote indigenous community in northern Canada.

**Summary Points**

- In many northern indigenous communities in Canada, systemic health disparities are compounded by extreme geographic isolation and limited access to emergency services.
- In these settings, the initial management of health emergencies depends on the capacity of laypeople. Few studies have explored the effects of first aid training in these settings.
- This paper reports on a collaboration to develop, deliver, and study a community-based first response training program in a remote indigenous community.
- A focus on geographically and culturally relevant content, pedagogy, and evaluative methods may transform first response training into an important local capacity-building, public health and health promotion intervention.
- This project advances a model for first response education programs in isolated and resource-poor settings, and offers socio-cultural insights into the role of first response programs in these settings.

## Introduction

Indigenous people face poor health outcomes in comparison with the Canadian population [1],[2]. In remote indigenous communities, the health impacts of historical and systemic disadvantage are compounded by geographic isolation and limited access to services. This presents a critical challenge in managing time-sensitive medical emergencies in very remote settings. In the absence of local paramedical services, the management of health emergencies depends on the capacity of laypeople. These communities in Canada mirror a global phenomenon: not only do rural and low-income populations worldwide face a disproportionate burden of disease, they also face systemic barriers to accessing timely care [3],[4].

Here we report on the Sachigo Lake Wilderness Emergency Response Education Initiative (SLWEREI), a collaboration between physicians, first aid educators, researchers, and a remote indigenous community to develop and deliver a life supporting first aid (LSFA) program in northern Canada. This program integrates community-based participatory research (CBPR) methods with LSFA training, and advances a potentially scalable model for LSFA education and research programs in remote and underserviced communities.

## Background

### Aboriginal Health in Canada

Indigenous populations in Canada—including First Nations, Inuit, and Métis peoples—are over-represented among populations with the poorest health in Canada, with markedly elevated rates of diabetes, hypertension, obesity, addiction, infectious disease, and suicide [1],[2],[5]. Life expectancy at birth for First Nations people in 2000 was 8.1 years less for men and 5.5 years less for women in comparison with the Canadian population [5]. The lifetime risk of severe trauma among First Nations populations is nearly four times the Canadian average, and accounts for one-third of deaths [6],[7].

According to the Assembly of First Nations, an application of the United Nations Human Development Index to living conditions in many First Nations communities would place them 63rd worldwide—“or amongst Third World conditions” [8]. For decades, researchers have identified the severe inadequacy of effective health infrastructure in these settings, including the lack of potable water and safe housing [2]. Political leadership and researchers have called for improved health services through an emphasis on capacity building, cultural continuity, and self-determination [9]–[11].

### Life Supporting First Aid Training

In many remote communities without formal paramedical services, laypeople and bystanders provide all on-site emergency care. First aid training programs have been shown to provide skills to engage in health promotion and address critical health emergencies [12],[13]. The Red Cross identifies first aid among essential health promotion interventions [14]. The World Health Organization asserts, “Even in settings with limited resources, many lives may be saved and disabilities prevented by teaching motivated people what to do at the scene.” [3]

LSFA training may confer public health benefits to populations with elevated rates of cardiac arrest and trauma [12],[15]. Cardiopulmonary resuscitation (CPR) performed by bystanders in urban settings may reduce sudden cardiac arrest mortality by as much as 22% [16],[17]. Basic pre-hospital services may reduce trauma mortality by 15%–20% [18],[19]. There is no strong evidence to support a singular educational or clinical approach to first aid in low-resource environments [20]. Compelling studies have demonstrated benefits arising from LSFA programs in remote and underserviced settings (Box 1).

**Box 1. LSFA in Remote and Underserviced Settings**

**Ghana:** Tiska et al. report on a first aid course for truck drivers in a low-resource setting with elevated rates of motor-vehicle trauma. The course emphasized airway management and external hemorrhage control, and improved the pre-hospital provision of first aid [25].

**Cambodia and Northern Iraq:** Husum et al. report on a 5-year prospective study to test a model for rural, low-income trauma services. More than 5,000 laypeople and paramedics were trained. Trauma mortality was reduced from 40% to 14.9%. Husum et al. conclude that training programs in isolated, low-resource settings can have significant mortality benefit, even with severely injured patients [26],[27].

**Uganda:** Jayaraman et al. report on a program to provide over 300 police officers, taxi drivers, and community leaders with a 1-day first aid course. Six months following the course, 97% of participants had used at least one skill learned in the course, and there was evidence of knowledge retention [28].

## The Project

### The Community and Setting

**Figure 1:**
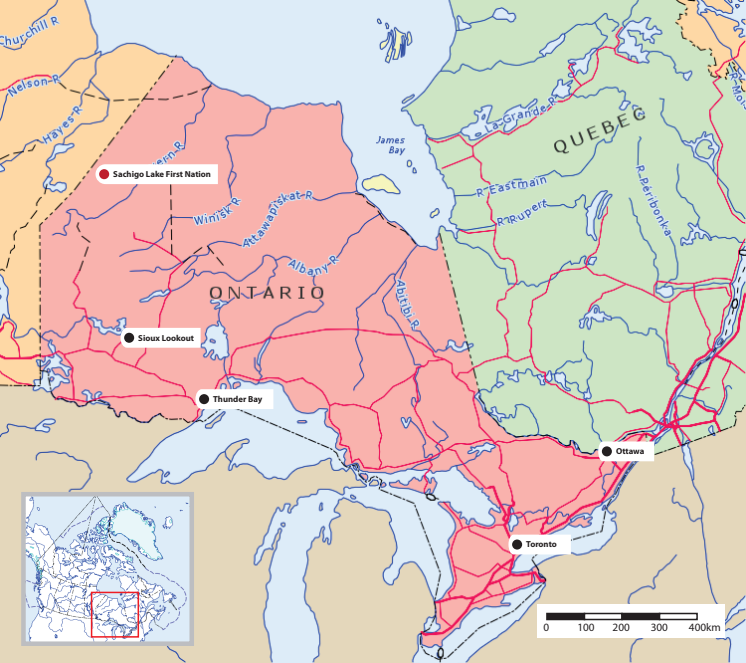
Map of Ontario showing Sachigo Lake First Nation. (Image: Department of Natural Resources Canada. All rights reserved).

Sachigo Lake First Nation (population 400) is a remote community in northern Canada (Figure 1). Similar to more than a hundred communities across Canada, Sachigo Lake is accessible only by air or seasonal ice roads. Full-time nurses and community health workers staff the local nursing station, funded by the Canadian government. A family physician visits the community for 2–3 days per month. Hospital care is provided hundreds of kilometres away, with transport times seldom less than 4 hours. Community members hunt, fish, and trap on their traditional lands, often travelling hours or days by motorboat, all-terrain vehicle, or snowmobile.

### The Collaboration

SLWEREI is a collaboration between Sachigo Lake First Nation, university researchers, and low-resource medicine educators. Our team involved three physicians and a paramedic experienced with low-resource medicine education, a researcher trained in CBPR, and community health leaders in Sachigo Lake.

CBPR transforms research into a vehicle for community engagement, and is an effective approach to the challenges of conducting research with marginalized populations [21]. CBPR can “enhance understanding of a given phenomenon and the social and cultural dynamics of the community, and integrate the knowledge gained with action to improve the health and well-being of community members.” [22]. CBPR may redress inequities and establish trust between communities and researchers [23],[24].

Our project methodology integrated community partners in every phase of development, delivery, and evaluation of the initiative. We developed a research agreement that emphasized equitable and reciprocal partnerships; sensitivity to Sachigo Lake community priorities; integration of programmatic and evaluative components; a flexible and responsive agenda; and the creation of a project representing learning opportunities for everyone involved. Community partners identified the program's effects on community resilience as a priority for evaluation. A successful program was described by community partners as one that created community satisfaction and engagement, and that enhanced the sense that emergencies could be managed appropriately. Community partners favoured participant observation and focus groups as the data collection methods.

The project team met with community stakeholders involved in governance and health care to discuss existing emergency systems, critical incidents, community perceptions about emergencies, and local training. Recent incidents had included motor vehicle and aircraft crashes, chainsaw and construction injuries, inhalational injuries, a near drowning, burns and frostbite, diabetic emergencies, myocardial infarction and strokes, suicide attempts, and aggressive behaviour. Community members articulated an interest in learning to adapt best practices in pre-hospital emergency care to the local context, rather than emphasizing skills that required new technologies or infrastructure.

### The Course and Data Collection

In November 2010, researchers and course instructors travelled to Sachigo Lake First Nation and coordinated an intensive 5-day LSFA training program, based on a curriculum and pedagogical approach designed specifically for the community. The community research partner selected the adult participants from various community roles, including community health workers, Canadian Rangers, school staff, maintenance and sanitation workers, local government, and general store employees. There were 20 course participants (5% of the community population, 13 men and seven women), including three community research partners.

**Figure 2:**
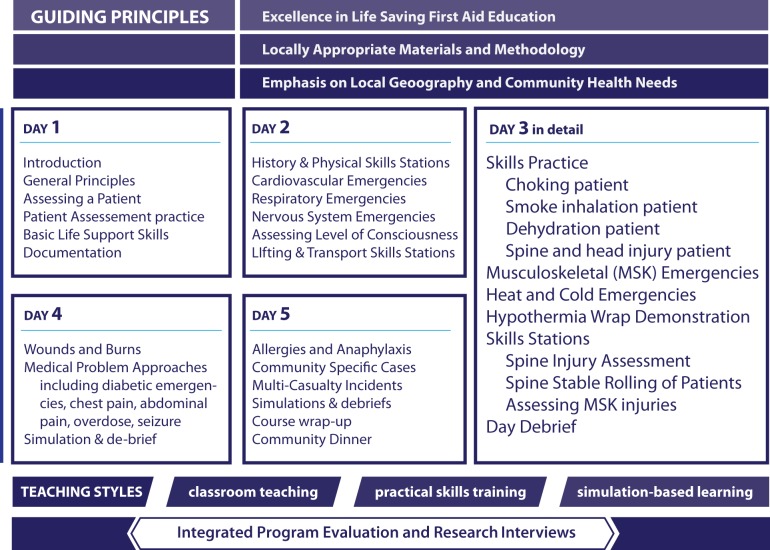
Schematic of life-supporting first aid training curriculum, evaluation, and pedagogical elements.

The curriculum focused on the immediate management and transportation of patients with critical health problems (Figure 2). The course included classroom teaching and discussions, small group skill-building exercises, and simulated emergency scenarios, but varied from conventional first aid programs by including opportunities for debrief, open question-and-answer sessions, and discussion about local challenges and experiences related to the medical problems being discussed.

Our course also integrated research, group reflection, and program evaluation. The course began with a plain-language informed consent presentation and discussion. Eight focus groups, with four to five participants each, as well as a sharing circle with all participants (*n* = 20) were integrated into the course. A semi-structured interview guide was used during focus groups. Community participants chose not to have focus groups recorded. A researcher took detailed notes during focus groups, interviews, and participant observation. Themes emerging from focus groups were discussed and validated with three members of the group who were selected by the community research partner. Daily feedback from the researcher to course instructors permitted course curriculum and pedagogy to be redirected and refined on the basis of participant concerns on a day-to-day basis.

## Results

A number of themes emerging from this research have implications for LSFA programs in remote and underserviced communities.

First, conventional first aid courses, their clinical content, and pedagogical assumptions may not meet the needs of remote communities. Some community members who had participated in conventional or standardized first aid training articulated dissatisfaction with courses conducted outside their cultural and geographic context. For example, where conventional first aid courses make the implicit assumption that first responders will likely provide care to strangers, Sachigo Lake community members have personal or familial experience with resuscitations, and expect to provide care to family and friends. One course participant remarked, “I had to splint up my daughter's arm last week. They [children] are rough with each other nowadays.” Another member of the group added that “from what I've seen from my own experience the kids are always putting stuff in their mouth. I've seen one kid choke already… I want to know what to do in that situation.” A course tailored to specific medical and cultural needs focused on providing first aid to family and friends, a unique feature when providing first response in a small community.

Second, LSFA education must be relevant to local communities and geographies. One community member relayed, “We … need training on how properly to transport patients, whether by boat … or snow machine.” Participants emphasized emergencies arising on hunting and trapping excursions: “We don't have a nursing station out there in the bush.” Another shared, “The scenarios … made me realize that it could actually happen to me if I need to help someone. …When I did [previous training courses] it didn't really click in with me.…This course was set in an environment that it might happen. … I took it to heart with this type of training…” While conventional urban first aid courses focus on immediate stabilization and activation of professional paramedical services, our curriculum involved prolonged patient care, and improvised equipment, wilderness terrain, and inclement weather. Simulation-based education reinforced principles learned in classroom and small group sessions, and brought real-world situations to life in an accessible way for all participants, regardless of educational background.

Finally, local participants identified local LFSA training as an important public health and health promotion intervention. Participants identified the longitudinal integration of evaluation, intensive debriefing, and open question-and-answer sessions as important and engaging parts of the program. Combining evaluation and reflection with skills training and practice enhanced a sense of community capacity and growth. Through this approach, not only does LSFA increase confidence in individuals, but also builds community resilience for remote populations. One participant remarked, “I know that there are people spread out across the community. I can call someone closer to respond.” Another indicated, “We're all going to benefit from it—not only the participants, but the general public from our community.”

## Discussion

Our work has convinced us that teaching urban, “standard” first aid in a remote and underserviced setting may deny those populations the skills needed to optimize outcomes or address medical emergencies with appropriate skills and confidence. First aid courses designed for temporary wilderness work or recreation may also be unsuitable for remote populations, and may assume a range of perspectives not present in isolated communities. Delivering first aid courses for remote communities involves re-thinking given notions of wilderness and isolation, especially where wilderness discourses and imagery suggest that life away from an urban tertiary care facility is inherently dangerous. Standard first aid may be an oxymoron: effective basic life support requires adaptation to local clinical, infrastructural, and cultural needs [20].

We found that insights from community members are important to ensure that course design and materials are relevant and sensitive to context. CBPR provides some guidance to engage members of the community and to validate and revisit assumptions drawn from focus groups. Where the “researcher-participant” relationship has long served the needs of the researcher, community-based and participatory health programming and research may contribute to a broad sense of community resilience and local capacity.

SLWEREI trained 5% of the Sachigo Lake population—an intervention comparable to training about 120,000 people in Toronto, Canada's largest city. In a community with no formal paramedical care, this may have an enormous impact on the management of health emergencies.

Our undertaking had limitations. Qualitative research design offers important socio-cultural insights and perspectives on community resilience, but cannot reveal morbidity or mortality effects. Like other similar public health interventions, designing a study to measure morbidity and mortality will be challenging considering the number of confounding variables, and the sample size of critical incidents in a community of 400 people. The public health and capacity-building effects are at once this project's most important outcome and its most challenging aspect to measure. By returning to the community in the future, we will gain a more in-depth and nuanced understanding of the public health impact of training 5% of a remote community in life-supporting first aid. Expansion beyond a single community may permit quantitative measurement of morbidity and mortality effects, and determine whether lessons learned in Sachigo Lake can be translated to other communities. Isolation defines the initiative, but also challenges effective collaborations, and is therefore an additional limitation. Transportation and shipping costs accounted for nearly 30% of the program budget.

## Future Directions and Conclusions

This project offers a novel collaborative approach to LSFA training in remote settings. Local and regional First Nations leaders have articulated an interest in sustaining and expanding the program. In other remote and low-resource communities worldwide, where bystanders and laypeople attend to the immediate needs of patients facing health emergencies, the lessons learned in Sachigo Lake may enhance local emergency first response capacity. A second course will be delivered in Sachigo Lake in 2012 to reinforce and refine teaching strategies, and curriculum. The course curriculum will be refined on the basis of participant and instructor feedback, including new mental health modules. The collaboration will expand to involve medical trainees from the Northern Ontario School of Medicine. Our team plans to explore opportunities to expand this model for community-specific first response training programs to other remote communities in Canada or abroad.

Context and community-specific LSFA training may contribute not only to patient outcomes in medical emergencies, but may also develop participant self-confidence and contribute to community resilience. Developing first response training programs in partnership with target communities and integrating longitudinal evaluation and community reflection into training curricula may further enhance these effects. LSFA education, developed and delivered with community collaboration, may provide a beneficial local health promotion intervention in remote and underserviced settings.

## Abbreviations

CBPR: community-based participatory research
LSFA: life supporting first aid
SLWEREI: Sachigo Lake Wilderness Emergency Response Education Initiative
